# Comparing the Feeding Damage of the Invasive Brown Marmorated Stink Bug to a Native Stink Bug and Leaffooted Bug on California Pistachios

**DOI:** 10.3390/insects11100688

**Published:** 2020-10-12

**Authors:** Judith M. Stahl, Davide Scaccini, Alberto Pozzebon, Kent M. Daane

**Affiliations:** 1 Department of Environmental Science, Policy & Management, University of California Berkeley, Mulford Hall, Berkeley, CA 94720, USA; davide.scaccini@phd.unipd.it (D.S.); kmdaane@berkeley.edu (K.M.D.); 2 Department of Agronomy Food Natural Resources Animals and Environment, University of Padova, Legnaro, 35020 Padova, Italy; alberto.pozzebon@unipd.it

**Keywords:** *Chinavia hilaris*, epicarp lesion, flat green stink bug, *Halyomorpha halys*, insect pest damage, leaffooted bug, kernel necrosis, *Leptoglossus zonatus*, stigmatomycosis

## Abstract

**Simple Summary:**

The brown marmorated stink bug is native to Asia and has invaded parts of Europe and North America where it causes considerable damage to a wide range of vegetable, fruit, and nut crops. Pistachios have become an important nut crop in California, and as this invasive stink bug moved into California, farmers needed to know about the potential damage from brown marmorated stink bugs. Here, we assessed pistachio yield loss and nut damage over a two-year period. The invasive stink bug was also compared with the native green stink bug and a native leaffooted bug. We found that brown marmorated stink bug adults cause more epicarp lesions (external damage) when recorded at harvest time than the native species; however, they did not cause more kernel necrosis (internal damage) than the two native species tested, which is a more relevant damage criterion for commercial production. We conclude that the brown marmorated stink bug could cause similar damage as the native species but note that the invasive stink bug’s numbers in California are still low and future damage levels will be dependent on this pest’s population density.

**Abstract:**

California currently produces about a quarter of the world’s pistachios. Pistachio nuts are susceptible to feeding by stink bugs and leaffooted bugs; therefore, the invasive presence of the highly polyphagous brown marmorated stink bug, *Halyomorpha halys* (Stål) (Hemiptera: Pentatomidae), is a concern to California pistachio growers. We aimed to assess the potential of *H. halys* to cause yield loss and nut damage to pistachios, which had not yet been assessed in the field. Over two years, terminal branch ends with pistachio clusters were enclosed in organdy cages from spring to fall and exposed to either *H. halys*, the native stink bug *Chinavia hilaris* Say (Hemiptera: Pentatomidae), or leaffooted bug *Leptoglossus zonatus* (Dallas) (Hemiptera: Coreidae), for 4–7-day feeding periods at different times of the season. We found that *H. halys* adults cause more epicarp lesions (external damage) when recorded at harvest time than the native species. They did not, however, cause more kernel necrosis (internal damage) than the two native species tested, which is a more relevant damage criterion for commercial production. There were no differences among insect species for any other recorded damage criteria. We conclude that *H. halys* could cause similar damage as the native species but note that *H. halys* population densities in California are still low and future damage levels will be dependent on this pest’s population density.

## 1. Introduction

Pistachios (*Pistacia vera* L.) have over 264,000 bearing acres (106,837 ha) in California, which produce about a quarter of the world’s pistachios [1]. As a crop, pistachio has a relatively short history in California, with commercial production rapidly increasing in the 1970s with the planting of the new cultivar ‘Kerman’ [2]; for this reason, many aspects of its production, including integrated pest management, are still being optimized. The most important insect pest in Californian pistachio orchards is the navel orangeworm *Amyelois transitella* (Walker) (Lepidoptera: Pyralidae) [3], but there is also a complex of heteropteran pests including Miridae and Rhopalidae (often referred to as ‘small bugs’), as well as Pentatomidae (stink bugs) and Coreidae (leaffooted bugs), that can be grouped together as ‘large bugs’, which can cause significant yield reductions especially in organic production [4,5].

**Figure 1:**
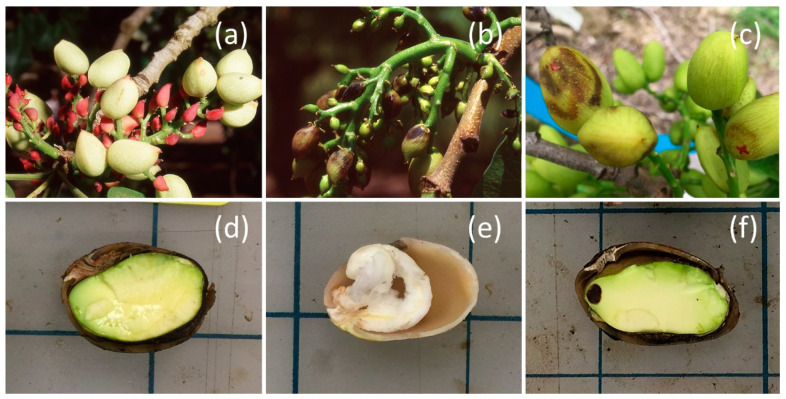
Pistachio clusters naturally drop nuts during fruit set (**a**), but heteropteran feeding can cause small nuts to blacken and drop (**b**) and larger nuts to form epicarp lesions (**b**,**c**); near harvest time, compared with an undamaged nut (**d**), heteropteran feeding can result in aborted nuts or blanks (**e**) and direct nut damage or kernel necrosis (**f**). Photos: Stahl and Daane.

Heteroptera cause crop damage by inserting their piercing—sucking mouthparts into the plant tissue, and either employing a cell-rupturing strategy or, using a salivary sheath, feeding on vascular tissue [6,7]. Some species also inject saliva with digestive enzymes that can cause discoloration, deformation, and abortion of fruiting structures [8,9,10]. In pistachios, damage by heteropterans may initially manifest as external damage with brown to black lesions of the outer fruit layer, called epicarp lesions (Figure 1b,c), which can also affect the shell’s meso- and endocarp tissue [11]. Direct internal damage shows as aborted nuts, especially prevalent while the endosperm is still developing, or necrotic tissue of the endosperm, kernel necrosis [4] (Figure 1e,f). Further damage is caused by contamination with fungi such as the yeast-like *Eremothecium coryli* Kurtzman (Saccharomycetales: Saccharomycetaceae) and *Aureobasidium pullulans* Arnaud (Dothideales: Dothioraceae) through the wound opening that can lead to stigmatomycosis [12,13]. The type and severity of pistachio damage from heteropteran feeding depend largely on the insect size and thereby the length of its mouthparts, and the physiological maturation of the fruit [4,14]. After the pistachio fruit sets until about mid-May, there is a period of natural nut drop (Figure 1a), and the tree can compensate for damaged nuts by reducing its natural nut drop [4]. In June, the pistachio shell hardens, which inhibits small bug feeding because of their smaller, weaker mouthparts, and may encumber large bug feeding [4]. Common large bug pests in Californian pistachio production include the stink bugs *Thyanta pallidovirens* (Stål), *Chlorochroa sayi* Stål, and *C. uhleri* (Stål), as well as *Chinavia hilaris* (Say) (formerly *Acrosternum hilare*) (Hemiptera: Pentatomidae), the leaffooted bugs *Leptoglossus zonatus* (Dallas), and *L. clypealis* Heidemann (Hemiptera: Coreidae). They overwinter as adults either inside the pistachio orchards or migrate into orchards in spring or summer [5,15]. In California’s pistachio production regions, stink bugs normally have two and leaffooted bugs up to three generations per year [15,16,17]. When large overwintering populations migrate into an orchard, naturally occurring biological control mainly from egg parasitoids may not be timely enough to suppress pest populations and insecticide application with pyrethroids or pyrethrin can become necessary [3].

One pentatomid species that has received a lot of attention during the past decade is the brown marmorated stink bug, *Halyomorpha halys* (Stål), which has invaded North and South America as well as Europe [18]. In the mid-Atlantic region of the USA, this pest caused a 37 million-dollar loss in apple production in 2010 alone [19]. The highly polyphagous *H. halys* feeds on many fruit crops, including vegetables, ornamentals, and nut crops [18]. On hazelnuts in Georgia, Italy, and Oregon, (USA) damage by *H. halys* has been substantial [20,21], and for this reason, it was believed that California nut crops were also in jeopardy after *H. halys* was detected in California in 2002 [16,22] and later reported in commercial peach and almond orchards [23,24]. To date, there has been no report of *H. halys* in commercial pistachio orchards, but laboratory studies have shown that *H. halys* accepts pistachio nuts as food and feeding causes necrotic kernels [22]. This study aims to determine the potential impact of *H. halys* on pistachio production by (1) determining the seasonal amount and type of nut damage and (2) comparing *H. halys* damage to that caused by the native *C. hilaris* and *L. zonatus*.

## 2. Materials and Methods

### 2.1. Insect Collection and Rearing

The *H. halys* colony was established in 2016 from individuals collected in Sacramento County, California, USA, the *C. hilaris* colony in 2017 from individuals in Fresno County, California, USA, and the *L. zonatus* colony in 2015 from individuals in Fresno County, California, USA. Groups of fifty adults each were maintained in the laboratory in organdy cages (BugDorm-4090, MegaView Science Co. Ltd., Taichung, Taiwan) at 25 °C, 35% RH, and a 16 L:8 D photoperiod. *Halyomorpha halys* and *C. hilaris* nymphs were moved to cages containing nymphs after they had reached the second instar to avoid cannibalism of egg masses. Once the nymphs had molted into adults, they were transferred into ‘adult’ cages. Food provided to all cages was a combination of store-bought fresh corn cobs (*Zea mays* L.), beans (*Phaseolus vulgaris* L.), carrots (*Daucus carota* subsp. *sativus* (Hoffm.) Schübl. & Martens), and zucchini (*Cucurbita pepo* L.); food was replaced twice per week. Greenhouse-grown potted bean plants of variable age that were grown in Miracle-Gro Potting Mix (The Scotts Company LLC, Marysville, OH, USA) were provided as an additional food source and as an oviposition substrate. Cages with *L. zonatus* were similarly provisioned with food; for oviposition, potted Mediterranean cypress (*Cupressus sempervirens* L.), and bamboo skewers (Best Brands Consumer Products, Inc., New York, NY, USA) placed vertically into Styrofoam boards were added.

### 2.2. Experimental Setup

Experiments were conducted in 2018 and 2019 in a 0.15 ha pistachio block irrigated with microsprinklers at the Kearney Agricultural Research and Extension Center near Parlier, California, USA. The block consisted of three pistachio varieties, ‘Kerman’, ‘Khaleghouchi’, and ‘Lost Hills’ planted fifteen years ago. No insecticide treatments were applied.

Each year, branches bearing pistachio clusters with a total of at least 20 nuts 1–2.5 m from the ground on the north side were covered with 5 gal (18.93 L) organdy cages (Paint Strainer Bags, Trimaco, Inc., Morrisville, NC, USA) before fruit set in April to exclude feeding damage by naturally occurring pests. Experiments were established in a complete randomized block design, with each tree functioning as a block and with enclosed pistachio clusters as treatment replicates. Each treatment consisted of one insect (*H. halys*, *C. hilaris*, *L. zonatus* adult in 2019, additionally *H. halys* third instar nymphs in 2018) per cage; there was a no-insect control. Every tree, therefore, contained five (in 2018) or four (2019) cages with an initial cluster load of at least 20 nuts.

Over the course of spring and summer, randomly chosen *H. halys*, *C. hilaris*, and *L. zonatus* from the laboratory were transferred into cages of randomly chosen trees. *Halyomorpha halys* third instar nymphs were also tested in 2018. For biosafety and availability reasons, only male *H. halys* adults were used in 2019. After about 5 days (4–7 days, depending on the ambient temperature), the insects were removed, and each nut was categorized as having an (1) epicarp lesion, (2) dropped, and (3) no damage, after which the cages were resealed until harvest.

In 2018, cages were inoculated with insect treatments every 2 weeks, from May through August, totaling seven trials, with each trial composed of ten replicates per species (and stage for *H. halys*) and the no-insect control. Based on 2018 results, we used fewer treatment periods in 2019, with the timing of each better suited to capture changes in pistachio nut phenology. We initiated four monthly setups with twelve replicates each: before natural nut drop, 23–27 April (corresponding with 2018 setups 2–27 May and 17–23 May); after nut drop, 22–29 May (corresponding with 2018 setup 31 May–5 June); midseason, 19–23 June (corresponding with 2018 setups 14–19 June and 28 June–3 July); and after shell-hardening, 17–24 July (corresponding with 2018 setups 2–7 August and 30 August–4 September).

At harvest time in September, nut clusters were collected, and in 2019, dropped nuts were recorded and removed. Harvested clusters were stored at 0.5 °C for no more than twelve weeks before processing. Every nut was evaluated for external (epicarp with lesions, or without marks) and internal (meat with kernel necrosis or signs of fungal infection, or nut aborted before development could be completed) damage. In 2018, nuts dropped after the cages were resealed post exposure were counted as ‘aborted’ during this stage.

To correlate insect mouthpart length with external and internal damage, rostra of individuals from the rearing were measured: randomly chosen individuals (n = 13 for each species, and stage for *H. halys*) were freeze-killed at −80 °C and sized with a Nikon SMZ800 stereomicroscope with a scaled ocular (Tokyo, Japan).

### 2.3. Statistical Analyses

Data are presented as proportion affected nuts of the total number of nuts per cluster at the time of recording in mean ± SE. N is the number of tested clusters. External damage criteria were nuts with epicarp lesions recorded immediately after exposure and during harvest time (before harvest in 2019 and within a few days after harvest in 2018), as well as dropped nuts recorded immediately after exposure. The internal damage criteria of the more than 10,000 harvested nuts were the nuts with kernel necrosis, signs of fungal infection (such as stigmatomycosis), and aborted nuts.

Each damage criteria was analyzed on nut-level with ‘treatment’ (*H. halys*, *C. hilaris*, *L. zonatus*, and the no-insect control), ‘pistachio phenology’ (before nut drop, after nut drop, midseason, and after shell-hardening), and ‘pistachio variety’ (Kerman, Khaleghouchi, and Lost Hills), as well as their interactions with ‘treatment’ as fixed effects and ‘cluster’ as a random effect in a generalized linear mixed model (GLMM) with a binomial error distribution. We checked the models for overdispersion and added an observation level random effect if necessary. We used backward simplification to generate models containing only significant fixed effect factors and their interactions. Only clusters with at least one nut were included in the analyses. Damage criteria were compared on nut-level between *H. halys* nymphs and adults with 2018 data with ‘treatment’ as a fixed effect and ‘cluster’ as a random effect in a GLMM with a binomial error distribution. To assess the mouthpart size, rostrum lengths were compared between insect species with an analysis of variance using a linear model.

Post-hoc pairwise comparisons of significant variables were conducted with a Tukey multiple comparisons test. All statistics were run with R version 3.6.2 [25] using RStudio version 1.2.5033 [26]. Packages used included ‘lme4’ [27] and ‘car’ [28] for the GLMM, as well as ‘multcomp’ [29], ‘multcompView’ [30], and ‘emmeans’ [31] for Tukey multiple comparisons.

## 3. Results

### 3.1. Epicarp Lesions Recorded Immediately after Insect Exposure

**Figure 2:**
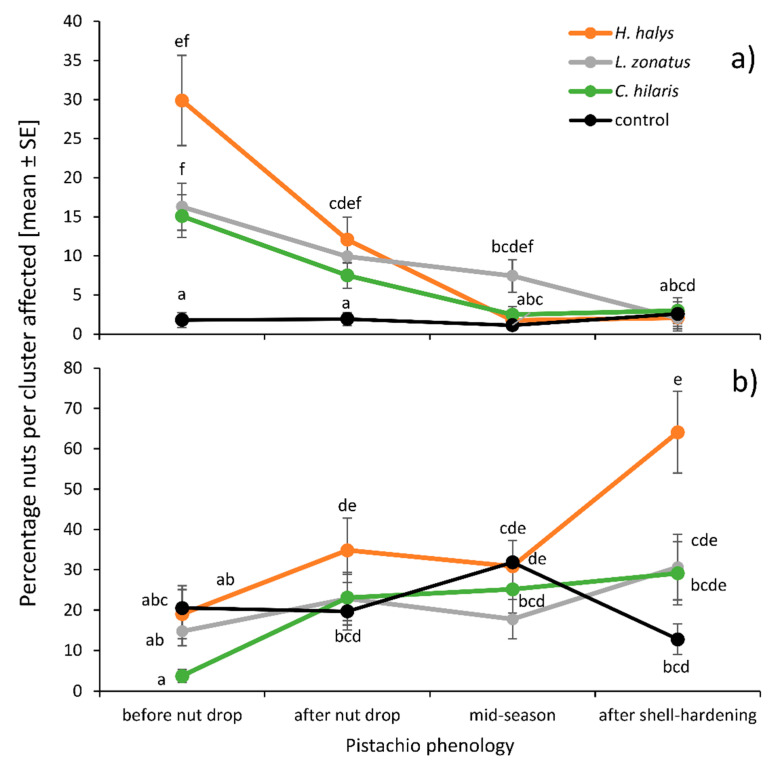
Interaction between insect treatment and pistachio phenology for (**a**) epicarp lesions immediately after insect exposure ‘el exp’ and (**b**) epicarp lesions during harvest time ‘el harv’. Different letters indicate significant differences (generalized linear mixed model (GLMM), Tukey-adjusted mean separations).

**Figure 3:**
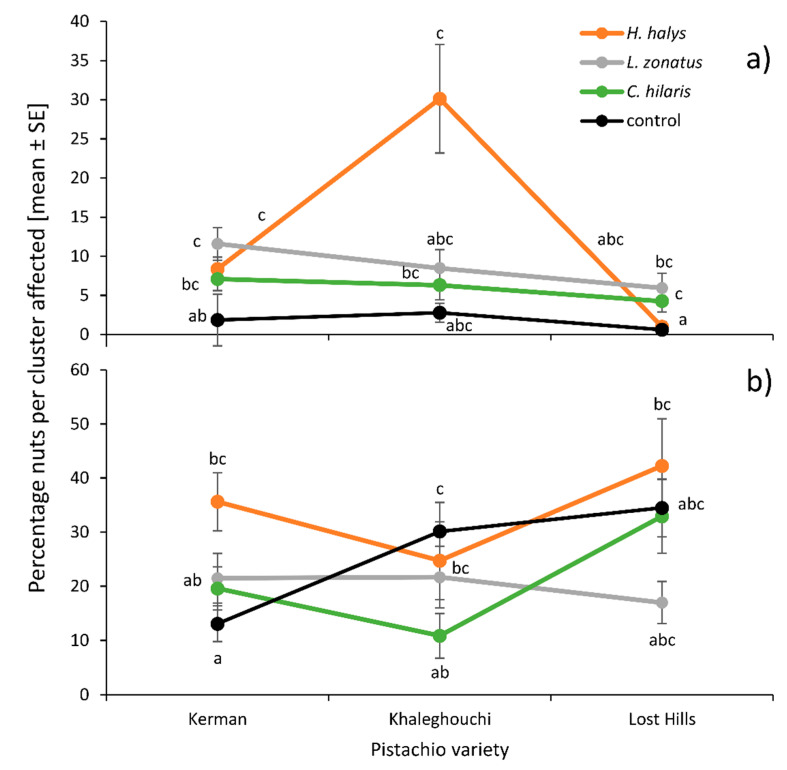
Interaction between insect treatment and pistachio variety for (**a**) epicarp lesions immediately after insect exposure ‘el exp’ and (**b**) epicarp lesions during harvest time ‘el harv’. Different letters indicate significant differences (GLMM, Tukey-adjusted mean separations).

**Figure 4:**
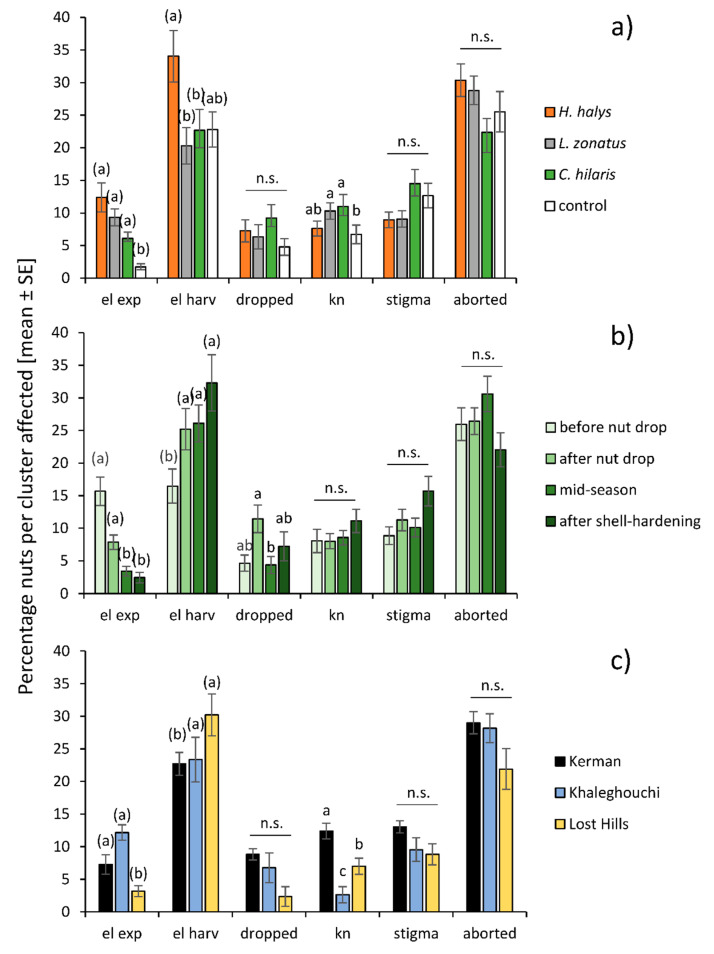
External and internal damage criteria recorded for (**a**) the different insect treatments, (**b**) the pistachio phenology, and (**c**) different pistachio varieties: ‘el exp’ = epicarp lesions recorded immediately after insect exposure, ‘el harv’ = epicarp lesions recorded during harvest, ‘dropped’ = dropped nuts, ‘kn’ = kernel necrosis, ‘stigma’ = fungal symptoms indicating stigmatomycosis, ‘aborted’ = aborted nuts. Treatments were compared within damage criteria for each part of the figure; bars with different letters are significantly different, comparisons without significant differences are headed with ‘n.s.’ (GLMM, Tukey-adjusted mean separations). Letters in parenthesis indicate an involvement in significant interactions (see Figure 2 and Figure 3).

The proportion of nuts showing epicarp lesions immediately after a 4–7-day feeding period ranged from 0% to 91.30% per cluster. The interaction between insect species and pistachio phenology was significant (GLMM, df = 9, 13627, *χ*^2^ = 39.06, *p* < 0.001): *Leptoglossus zonatus* produced more epicarp lesions during ‘midseason’ than the other two species, but the trend of decreasing epicarp lesions with the progressing season was similar for all insect species (Figure 2a). The interaction between insect species and pistachio variety was significant (df = 6, 13627, *χ*^2^ = 20.234, *p* = 0.0025) because only *H. halys* seemed to produce more epicarp lesions on the ‘Khaleghouchi’ (30.13% ± 7.00, n = 19) variety than on ‘Kerman’ (8.36% ± 1.54, n = 47) or ‘Lost Hills’ (0.99% ± 0.76, n = 10), even though this was not significant (Tukey multiple comparisons *p* > 0.05), likely due to the low and unbalanced sample size (Figure 3a). When considering the fixed effects individually, *H. halys* produced similar numbers of epicarp lesions (12.38% ± 2.21, n = 80) as *L. zonatus* (9.35% ± 1.29, n = 91) and *C. hilaris* (6.10% ± 0.96, n = 70) (Figure 4a). All insect treatments were significantly higher than the no-insect control (1.73% ± 0.45, n = 91) (df = 3, 13627, *χ*^2^ = 63.50, *p* < 0.001) (Figure 4a). The most epicarp lesions were recorded when insects fed in the first half of the season, ‘before nut drop’ (15.96% ± 2.20, n = 80), followed by ‘after nut drop’ (7.85% ± 1.10, n = 84), while ‘midseason’ feeding (3.41% ± 0.73, n = 103) and exposure ‘after shell-hardening’ (2.43% ± 0.788, n = 60) produced fewer epicarp lesions (Figure 4b). Pistachio variety had no effect (df = 2, 9597, *χ*^2^ = 4.81, *p* = 0.0903), but ‘Lost Hills’ showed significantly fewer (3.17% ± 0.72, n = 83) epicarp lesions than ‘Kerman’ (7.26% ± 0.81, n = 177) and ‘Khaleghouchi’ (12.17% ± 2.35, n = 73) (Figure 4c).

### 3.2. Epicarp Lesions Recorded During Harvest Time

The proportion of nuts showing epicarp lesions during harvest time ranged from 0% to 100% per cluster. The interaction between insect species and pistachio phenology was significant (df = 9, 13560, *χ*^2^ = 20.14, *p* = 0.0013) with *H. halys* causing more epicarp lesions when feeding toward the end of the season in comparison to feeding at the beginning, while the other species did not show this trend as clearly (Figure 2b). The interaction between insect species and pistachio variety was significant (df = 6, 13560, *χ*^2^ = 21.82, *p* = 0.0171) because *L. zonatus* produced the least amount of epicarp lesions on ‘Lost Hills’, and both *H. halys* and *C. hilaris* on ‘Khaleghouchi’, but none of those trends were significant (Figure 3b). Overall, *H. halys* produced more epicarp lesions (34.05% ± 3.99, n = 80) than *L. zonatus* (20.30% ± 2.89, n = 94) and *C. hilaris* (22.70% ± 3.33, n = 71) but not the no-insect control (22.78% ± 2.73, n = 89) (df = 3, 13562, *χ*^2^ = 9.05, *p* = 0.029) (Figure 4a). The least epicarp lesions at harvest time were recorded after insect feeding ‘before natural nut drop’ (16.48% ± 2.75, n = 79), while feeding later in the season, ‘after nut drop’ (25.21% ± 3.22, n = 87), as well as ‘midseason’ (26.08% ± 2.812, n = 108) and ‘after shell hardening’ (32.30% ± 4.44, n = 60), produced more nuts with lesions (Figure 4b). The ‘Kerman’ variety showed significantly fewer (22.70% ± 2.39, n = 165) epicarp lesions than ‘Khaleghouchi’ (23.34% ± 3.13, n = 71) and ‘Lost Hills’ (30.22% ± 3.07, n = 83) (Figure 4c).

### 3.3. Dropped Nuts

The proportion of dropped nuts ranged from 0% to 100% per cluster. No interactions were significant. The insect treatments were not significantly different from each other (Tukey multiple comparisons, *p* > 0.05) (Figure 4a). Neither the beginning of the season (before natural nut drop: 4.64% ± 1.26, n = 80) nor the end of the season (after shell-hardening) (7.22% ± 1.31, n = 60) were different from any other time point, but significantly more nuts dropped immediately after exposure when insects fed ‘after nut drop’ (11.44% ± 2.12, n = 85) than shortly thereafter during ‘midseason’ (4.37% ± 0.15, n = 108) (Figure 4b). The pistachio variety had no effect on the number of dropped nuts (df = 2, 13562, *χ*^2^ = 2.99, *p* = 0.2243) (Figure 4c).

### 3.4. Kernel Necrosis

The proportion of nuts showing kernel necrosis ranged from 0% to 76.92% per cluster. No interactions were significant. *Leptoglossus zonatus* (10.30% ± 1.29, n = 90) and *C. hilaris* (11.02% ± 1.91, n = 61) produced more necrotic kernels than the no-insect controls (6.71% ± 1.42, n = 91). *Halyomorpha halys* (7.61% ± 1.18, n = 80) was not significantly different from the other treatments (df = 3, 9596, *χ*^2^ = 11.78, *p* = 0.0082) (Figure 4a). There was no influence of pistachio phenology (df = 2, 9596, *χ*^2^ = 1.42, *p* = 0.7011). Pistachio nuts of the ‘Khaleghouchi’ variety showed the fewest (2.61% ± 0.54, n = 73) necrotic kernels, followed by ‘Lost Hills’ (6.98% ± 0.93, n = 83), which showed less necrotic kernels than those of the ‘Kerman’ variety (12.39 ± 1.23, n = 169) (df = 2, 9596, *χ*^2^ = 34.23, *p* < 0.001) (Figure 4c).

### 3.5. Stigmatomycosis

The proportion of nuts with fungal symptoms ranged from 0% to 80.00% per cluster. No interactions were significant. There was no significant effect of insect treatment (df = 3, 9597, *χ*^2^ = 4.10, *p* = 0.2512) or pistachio variety (df = 2, 9597, *χ*^2^ = 5.05, *p* = 0.0799), and despite being recorded as a significant effect (df = 3, 9597, *χ*^2^ = 8.09, *p* = 0.0442), there were no differences between pistachio phenology time points (Tukey multiple comparisons, *p* > 0.05) (Figure 4b).

### 3.6. Aborted Nuts

The proportion of aborted nuts ranged from 0% to 100% per cluster. No interactions were significant. Insect treatment was significant in the GLMM (df = 3, 9597, *χ*^2^ = 8.29, *p* = 0.0403), but there were no differences between the treatments (Tukey multiple comparisons, *p* > 0.05) (Figure 4a). Neither pistachio phenology (df = 3, 9597, *χ*^2^ = 6.63, *p* = 0.0849) nor variety (df = 2, 9597, *χ*^2^ = 4.81, *p* = 0.0903) had an effect (Figure 4b,c).

### 3.7. Comparison between H. halys Adults and Nymphs

*Halyomorpha halys* nymphs caused significantly fewer epicarp lesions immediately after insect exposure than *H. halys* adults (df = 1, 3405, *χ*^2^ = 8.29, *p* = 0.0040), but more when lesions were recorded during harvest time (*χ*^2^ = 7.25, *p* = 0.0071). There were no differences between nymphs and adults for the number of dropped nuts per cluster (*χ*^2^ = 0.40, *p* = 0.5285), with necrotic kernels (*χ*^2^ = 0.38, *p* = 0.5364), or signs of stigmatomycosis (*χ*^2^ = 0.92, *p* = 0.3371). Adult feeding caused more aborted kernels than nymph feeding (*χ*^2^ = 8.12, *p* = 0.0044).

### 3.8. Rostrum Length

Rostrum length differed among adults of the three insect species (ANOVA, df = 2, 37, *F* = 331.12, *p* < 0.001), with *L. zonatus* having the longest rostrum (11.10 ± 0.20 mm, n = 13), *H. halys* an intermediate (6.95 ± 0.09 mm, n = 13), and *C. hilaris* the shortest rostrum (6.28 ± 0.12 mm, n = 14). When *H. halys* nymphs were included in the analysis (LM, df = 3, 48, *F* = 392.96, *p* < 0.001), they had significantly shorter rostra than *C. hilaris* (Tukey multiple comparisons, *t* = 11.015, *p* < 0.001).

## 4. Discussion

This first field study of *H. halys* in pistachios shows this invasive pest can cause damage to pistachio nuts throughout the season, resulting in epicarp lesions and kernel necrosis. This is consistent with the type and extent of damage caused by native stink bugs and leaffooted bugs [4], and confirms the formation of kernel necrosis from *H. halys* feeding, which was previously shown in a laboratory study [22]. There were seasonal differences in the type and amount of damage: whereas internal damage symptoms were not affected by the time of the season the insects were feeding, the formation of epicarp lesions was differentially impacted. When recorded immediately after insect exposure, most lesions appeared early in the season for all tested pest species. When recorded during harvest time, *H. halys* late-season feeding after shell-hardening caused more external damage than early-season feeding before natural nut drop, something that could not be confirmed for *C. hilaris* or *L. zonatus*. This was the only instance where we could see the previously reported effect that heteropteran feeding early in the season can be compensated during the natural nut drop in May [5,15].

Epicarp lesions are an external symptom of a feeding injury [14,32,33] and appear to form quickly when the nut epicarp is still growing early in the season and are more delayed when feeding occurs at a later phenological stage. Sometimes they do not form at all, leading to hidden damage [4,11]. After feeding by all insect species, we recorded an increase in epicarp lesions from directly after exposure to harvest time. Delayed symptoms are common for *H. halys* and native large bug feeding [34,35] and can obscure the association of damage symptoms with the perpetrator since the insects may have often long left the crop by the time damage becomes apparent [4].

Even without internal damage such as kernel necrosis, epicarp lesions can lower market value significantly if it causes the shell to be stained [5]. The number of epicarp lesions per cluster recorded directly after insect exposure varied differentially between years: while *H. halys* caused fewer epicarp lesions in 2019 than in 2018, the native *C. hilaris* and *L. zonatus* caused more epicarp lesions in 2019 (Figure S1). This is mainly driven by the exposure periods before natural nut drop and might be caused by differences in ambient temperatures and relative humidity during these exposures: in 2018, temperatures ranged from 12 °C to 30 °C and 28% to 72% RH, while in 2019, this period was characterized by overall higher temperatures (17 °C to 35 °C) and lower relative humidity (19% to 60%) [36]. These slight differences could have favored the native species and hindered *H. halys* for which prolonged periods of temperatures above 30 °C are detrimental [37,38,39,40]. After *H. halys* exposure, these epicarp lesions would affect around a third of the harvested nuts. The difference among the tested insect species might be based on differences in their saliva composition, or their feeding strategies [8]. So far, only the saliva of *H. halys* has been analyzed [41], but differences between *H. halys* and other Pentatomidae in terms of salivary enzyme activity have been recorded [42]. Those could be related to different feeding strategies. Hemipteran feeding strategies are divided into ‘cell-rupturing’ and ‘vascular feeding’, which includes a salivary sheath [43]. Stink bugs and leaffooted bugs, belonging to the infraorder Pentatomorpha, are rarely limited to a single feeding strategy [44]. Instead, they can alter their strategy with their development or adapt it to the respective plant tissue [44]. Recently, Serteyn et al. [7] discovered that *H. halys* utilizes both feeding strategies to potentially feed on xylem, phloem, and mesophyll/parenchyma, showing a uniquely high plasticity even for Pentatomorpha. This could, potentially, lead to a different damage pattern than *C. hilaris* and *L. zonatus*. Other factors that have been suggested to impact crop damage by stink bugs include the time the insects spend feeding [45], which is a variable we could not record in our field study.

A variable that could influence internal damage is, in combination with the shell-hardiness of the crop, the morphology of the mouthparts including the length of the insect stylet [46]. Furthermore, pentatomid species with a similar body size having a short rostrum may exhibit deeper stylet penetration potential than other species with a longer rostrum, likely due to mechanical features linked to the length of rostral segments [47]. Since there were significant differences in rostrum lengths of the three tested insect species, but not in the number of nuts per cluster with kernel necrosis, we conclude that the shortest measured adult rostra, those of *C. hilaris*, are long enough to pierce the pistachios even after shell hardening, and increased rostrum length above a certain threshold does not lead to more damage on pistachio nuts. *Halyomorpha halys* nymphs possessed the shortest rostrum and caused as much internal damage as *H. halys* adults except for aborted kernels, however, there were also no differences to the control, so conclusions are difficult to draw. The fact that, in the control clusters, both external, as well as internal damage was recorded showed that caging them early in the season to exclude herbivores was not fully successful and confounds the damage data to a certain extent. The three pistachio cultivars showed no differences in susceptibility to feeding by either *H. halys* or the two native species. The standard cultivar in California ‘Kerman’ overall showed the highest proportion of necrotic kernels, potentially indicating a higher susceptibility to large bug feeding damage. A larger sample size for the newly developed ‘Lost Hills’ variety and the Iranian cultivar ‘Khaleghouchi’ might have shed light on more potential differences.

Stigmatomycosis is a fungal infection of fruits, transmitted by hemipteran feeding that has been reported for pistachios both in Iran and California, USA [13,48]. In the present study, no differences in the number of nuts per cluster with stigmatomycosis could be found between the insect treatments and the no-insect control. This could indicate that the recorded fungal infections were not all caused by hemipteran feeding. Instead, the period of storage between harvest and processing could have led to increased decomposition of all nuts, masking the effect of the insect-induced stigmatomycosis. This is supported by the higher levels of fungal infections in 2019, which correlated with a longer storage period. However, in some crops, the presence of *H. halys* was shown to increase the occurrence of fungal infections on fruits [21,49,50], and these issues have to be taken into account considering the damage potential of this pest in particular on fruit crops. Dropped and aborted nuts showed no differences between treatments.

*Halyomorpha halys* nymphs caused a similar amount of damage as adults, apart from fewer aborted kernels. This is different from *C. hilaris*, whose second and third instar nymphs caused significantly less damage than their adult counterparts during midseason and only converge during the latter part of the season [4], indicating that *H. halys* nymphs could potentially be more harmful than the nymphs of *C. hilaris*.

Using more than one insect per pistachio cluster might have caused more damage [48] and revealed differences that now remain hidden, but one adult per cluster mirrors realistic conditions in a pistachio orchard. In contrast to adults, early instar nymphs of all three tested species often occur clustered after hatching from their egg mass but are generally dispersed by the third instar when they are more likely to cause damage [51,52]. However, *H. halys* as an invasive species is of concern to growers especially due to their high abundance in invaded areas. In Allentown, PA, where the first *H. halys* of the USA had been recorded, it has become the predominant stink bug species within twenty years [53].

## 5. Conclusions

Overall, *H. halys* causes the same amount and type of pistachio damage as *C. hilaris* and *L. zonatus*. However, whether *H. halys* will become a serious threat to Californian pistachio production will depend on its presence and abundance in the respective counties. Despite its highly invasive behavior in other parts of the country and the world [18], *H. halys* abundance in California has so far been surprisingly low, and some have suggested an impact of hot dry climate in the interior valleys may reduce its densities [16], whereas *L. zonatus* populations appear to be increasing in population density or survival [15,54]. Further studies looking into *H. halys* survival and potential limiting factors to its abundance in California are, therefore, necessary to make more informative predictions.

## Supplementary Materials

The following are available online at https://www.mdpi.com/2075-4450/11/10/688/s1, Figure S1: External and internal damage criteria recorded for the different insect treatments separated by year.
