# Supplementary material for: Comparing the Feeding Damage of the Invasive Brown Marmorated Stink Bug to a Native Stink Bug and Leaffooted Bug on California Pistachios
Source: Insects. 2020 Oct 12;11(10):688. doi: 10.3390/insects11100688 (PMC7599547; doi:10.3390/insects11100688)
Supplement: Supplementary file 1 [file insects-11-00688-s001.pdf]

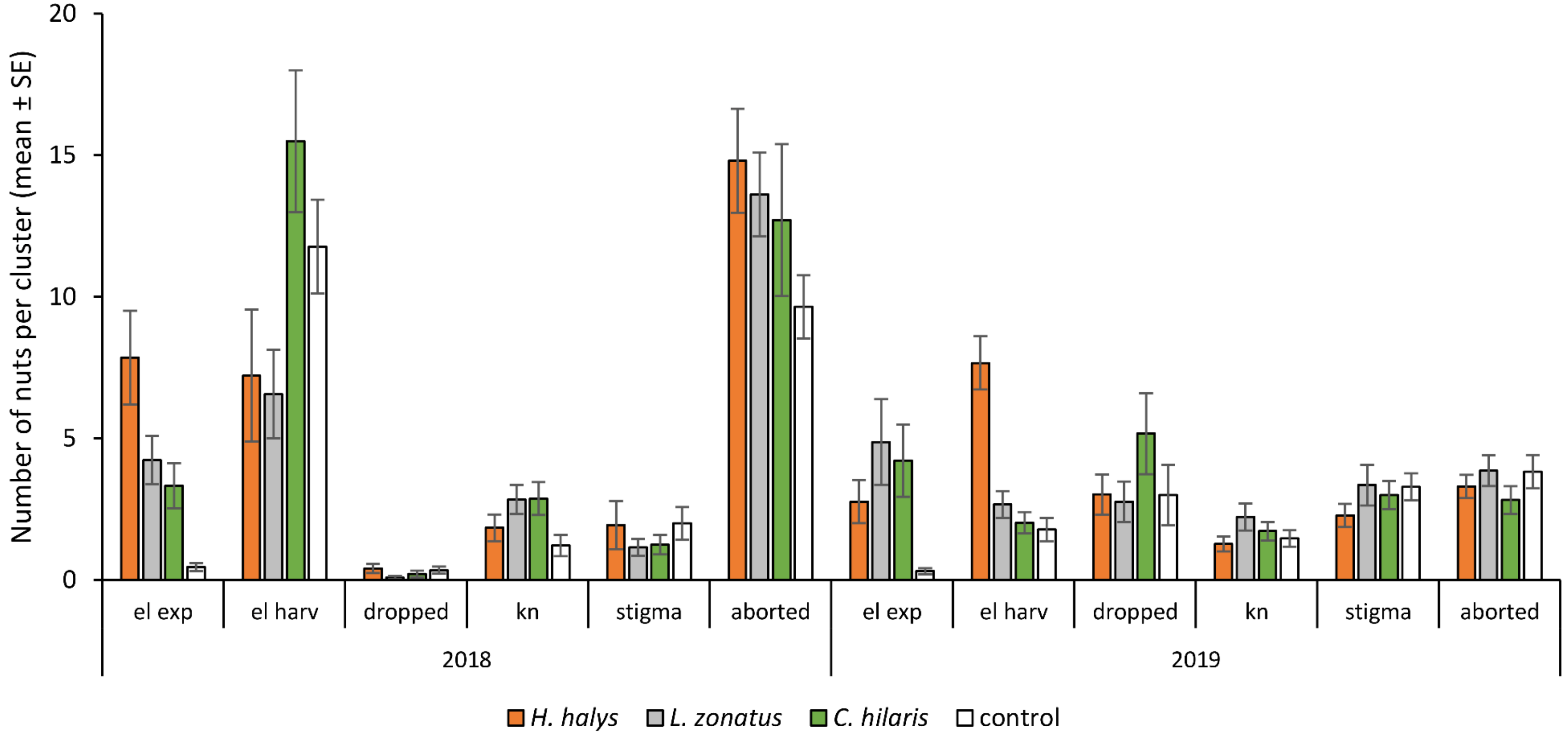

Figure S1: External and internal damage criteria recorded for the different insect treatments separated by year: 'el exp' = epicarp lesions recorded immediately after insect exposure, 'el harv' = epicarp lesions recorded during harvest, 'dropped' = dropped nuts, 'kn' = kernel necrosis, 'stigma' = fungal symptoms indicating stigmatomycosis, 'aborted' = aborted nuts. Differences between years in 'dropped' and 'aborted' nuts are likely due to a change in damage evaluation. Differences between years in 'el harv' nuts are likely due to temporal differences in data recording with recording in the laboratory after a period in storage of up to four weeks in 2018 and recording in the field in 2019.
